# Degradation of Anthraquinone Dye Reactive Blue 4 in Pyrite Ash Catalyzed Fenton Reaction

**DOI:** 10.1155/2014/234654

**Published:** 2014-01-09

**Authors:** Milena Becelic-Tomin, Bozo Dalmacija, Ljiljana Rajic, Dragana Tomasevic, Djurdja Kerkez, Malcolm Watson, Miljana Prica

**Affiliations:** ^1^Department of Chemistry, Biochemistry and Environmental Protection, Faculty of Sciences, University of Novi Sad, Trg Dositeja Obradovica 3, 21000 Novi Sad, Serbia; ^2^Faculty of Technical Sciences, University of Novi Sad, Trg Dositeja Obradovica 6, 21000 Novi Sad, Serbia

## Abstract

Pyrite ash (PA) is created by burning pyrite in the chemical production of sulphuric acid. The high concentration of iron oxide, mostly hematite, present in pyrite ash, gives the basis for its application as a source of catalytic iron in a modified Fenton process for anthraquinone dye reactive blue 4 (RB4) degradation. The effect of various operating variables such as catalyst and oxidant concentration, initial pH and RB4 concentration on the abatement of total organic carbon, and dye has been assessed in this study. Here we show that degradation of RB4 in the modified Fenton reaction was efficient under the following conditions: pH = 2.5; [PA]_0_ = 0.2 g L^−1^; [H_2_O_2_]_0_ = 5 mM and initial RB4 concentration up to 100 mg L^−1^. The pyrite ash Fenton reaction can overcome limitations observed from the classic Fenton reaction, such as the early termination of the Fenton reaction. Metal (Pb, Zn, and Cu) content of the solution after the process suggests that an additional treatment step is necessary to remove the remaining metals from the water. These results provide basic knowledge to better understand the modified, heterogeneous Fenton process and apply the PA Fenton reaction for the treatment of wastewaters which contains anthraquinone dyes.

## 1. Introduction

Pyrite is one of the most abundant sulphide minerals on the Earth containing high content of iron [1] and has been widely used in modern industry for the production of sulphuric acid. The hematite-rich waste, known as roasted pyrite ash, or pyrite ash, is left as residue after the following processes in the production of sulphuric acid: roasting for the pyrite concentrate; cooling of the gases in the pot, and purifying with dry electronic purifiers and cyclones [2]. This waste could be used in the environmentally safe manner, as an iron ore in the steel, brick, paint, and cement industries [3]. However, the iron oxide-rich residue contains significant concentrations of potentially polluting elements (e.g., Cu, S, Zn, Pb, and As) which can be very mobile under environmental conditions, reducing its applicability [2, 4]. In Serbia, sulphuric acid is dominantly made by pyrite processing. In the last couple of years, Serbia has disposed of 300–500,000 tons of pyrite ash in neglected landfills (http://www.ekoplan.gov.rs/). However, the landfill's capacity is limited, and were the waste contaminated, it could represent a serious threat to groundwaters, soils, and settlements.

Textile manufacturing is one of the largest industrial producers of wastewaters, which are characterized by strong colour, highly fluctuating pH, high chemical oxygen demand (COD), and biotoxicity. Reactive dyes are frequently used for dyeing cotton, wool, and polyamide fibres. Under typical reactive dyeing conditions, not all dyes bind to the fabric; depending on the class of dye, up to 50% of the initial dye remains in the spent dye bath in its hydrolyzed form, which has no affinity for the fabric and results in a coloured effluent. Reactive dyes are known to be nondegradable under the typical aerobic conditions found in conventional biological treatment systems and adsorb very poorly biological solids, resulting in residual colour in discharged effluents [5]. Although azo dyes represent about 60% of all reactive dyes used by the textile industry, other classes of reactive dyes, namely anthraquinone and phthalocyanine dyes, are also extensively used either as primary or secondary dyes in commercial trichromatic dyeing formulations [6, 7]. However, in sharp contrast to the considerable research that has been conducted on the biotransformation and decolourization of azo dyes, limited information exists on the reductive transformation of both anthraquinone and phthalocyanine reactive dyes. In particular, detailed information is lacking with regard to the behaviour of aqueous reactive anthraquinone dyes, as well as the application of advanced analytical/instrumental techniques for the identification and quantification of the parent compounds as well as the dye transformation products.

To eliminate dyes from aqueous coloured effluents and reduce their ecological consequences, several biological and chemical techniques have been proposed (adsorption, coagulation, and biological treatments). Traditional processes for treatment of these effluents prove to be insufficient to clean up the important quantity of wastewaters after different operations of textile dyeing and washing [8]. In recent years, promising results have been achieved in the treatment of textile wastewaters using advanced oxidation processes (AOPs) [5, 9–12]. A variety of AOPs are based on ozonolysis, UV/Vis photocatalysis and the Fenton reaction [1] and include the formation, and reactions of ^•^OH radicals that are much more reactive than all the other oxidizing species used in oxidative pollution abatement in drinking water and in wastewater [5, 13] These highly reactive radicals promote the destruction of the target pollutant until mineralization [14, 15]. The advantage of classic Fenton processes besides having a powerful oxidative capacity is that they neither transfer pollutants from one phase to the other nor produce massive amounts of hazardous sludge [16]. However, the classic Fenton reaction has a fatal limitation for remedial application due to the early termination of the degradation reaction by the rapid precipitation of iron hydroxide in circumneutral pH environments [16]. The disposal of ferric hydroxide sludge requires additional unit operations [17, 18].

Efforts to overcome the limitations of the classic Fenton reaction have been made by modifying the system and/or introducing novel alternative iron sources. Numerous researchers [19–22] have shown that H_2_O_2_ can oxidize organic pollutants in the presence of solid catalysts which contain iron. In the literature, these processes are known as “modified heterogeneous Fenton processes” [23, 24]. Iron oxides have been put forward as potential promising iron sources for the modified Fenton reaction to treat a variety of environmental contaminants in wastewater and groundwater systems [25]. However, to date, limited basic knowledge has been produced to characterize the pyrite ash in heterogeneous Fenton reaction for the development of enhanced remediation technologies to treat environmental contaminants, such as anthraquinone dyes.

If waste materials, such as pyrite ash, can be used in the modified Fenton process as an alternative source of catalytic material for anthraquinone dye degradation, it would be, at the same time, a cost-effective method for its disposal and minimise possible negative environmental effects originating from either the pyrite ash or wastewaters, through proper engineering control.

In view of the above, the objectives of this study were (1) to evaluate the effectiveness of the oxidative degradation and mineralization of reactive anthraquinone dye, reactive blue 4 (RB4), in aqueous solutions using pyrite ash, as a catalytic material in a modified heterogeneous Fenton process, (2) to evaluate the influence of operative conditions on dye degradation, (3) to evaluate the contribution from a homogenous Fenton reaction to the overall heterogeneous Fenton reaction, (4) to determine the residual metal content in the aqueous solution and (5) to evaluate the possibility of metal immobilization after the Fenton reaction.

## 2. Materials and Methods

**Figure 1:**
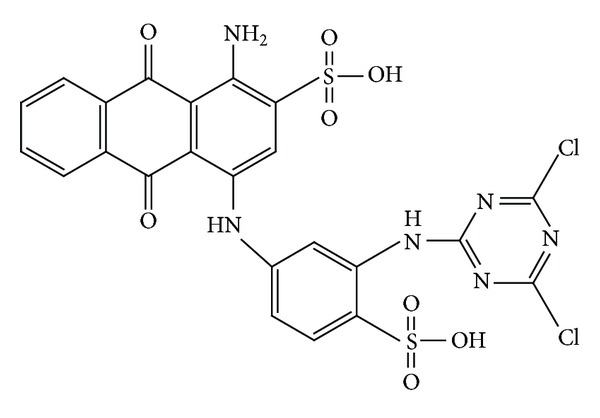
Structure of RB4 dye.

Commercial RB4 (CAS no. 13324-20-4, EC no. 236-363-6, and pH 4.78), H_2_O_2_ (30 wt.%), H_2_SO_4_, and quicklime (99% CaO) were of obtained from Sigma-Aldrich. All chemicals used were analytical grade and used without further purification. All solutions were prepared with deionised water. All experiments were conducted at room temperature (25 ± 0.5°C).  Figure 1 presents the structure of the dye investigated.

The PA used in this work was left after the combustion of pyrite in the production of sulphuric acid in the Chemical Industry “Zorka” Sabac. The chemical composition of the PA (wt. > 1%) was determined by wavelength dispersive X-ray fluorescence spectrometry (S8 Tiger, Bruker, Germany): hematite 79.74%, quartz 9.09%, magnetite 5.08%, calcite and 1.36%, plagioclase 1.21%.

PA was washed three times in double distilled water prior to conducting the experiment and was dried for an hour at 105°C. The PA digestion for assessing the metals concentrations was conducted in a Milestone Start E (Milestone, Germany) microwave oven [26]. Mean values of metals were used: 913.8 ± 52 mg kg^−1^ (Zn), 106.2 ± 5.8 mg kg^−1^ (Cr), 572.8 ± 33 mg kg^−1^ (As), 2130 ± 154 mg kg^−1^ (Pb), 5227 ± 345 mg kg^−1^ (Fe), 1492 ± 110 mg kg^−1^ (Cu), and 18.9 ± 0.8 mg kg^−1^ (Cd). The relative standard deviations (% RSD) obtained (*n* = 3) were below 10%.

The metals content in the PA was determined by atomic absorption spectrophotometer (AAS) Perkin Elmer Analyst 700 using standard methods [27, 28].

The experiments were conducted on a jar test apparatus (FC6S Velp Scientifica, Italy), where reactive mixtures with 0.25 L volumes were continually mixed in 1 L laboratory cups, at 150 rpm. The experiments were performed in the following manner: firstly, the appropriate PA dosage (0.05–0.5 g L^−1^) was added to the model dye solution (RB4 concentration from 50 to 250 mg L^−1^), which was followed by pH adjustment to the desired value (2.5–4) and the addition of the required amount of H_2_O_2_ (1.0–25 mM). Each experiment lasted 30 min. Samples were centrifuged at 12000 rpm for 5 min to remove solid particles. The aliquots were subsequently filtered through a 0.45 *μ*m pore size membrane filter and immediately analyzed thereafter.

Decolourization of the synthetic dye solution was monitored by absorbance (*A*) at *λ*_max_ = 594.78 nm (UV/VIS spectrophotometer, Shimadzu, Japan). Total organic carbon (TOC) concentrations were determined by Elementar Liqui TOCII analyzer, Germany. The pH was measured by pH-meter inoLap pH/ION 735 (WtW GmbH, Germany).

The efficiency of dye decolourization and oxidation was obtained by the application of the following formulas:

(1) $$\begin{array}{c} 100\left(\frac{A_{0}-A}{A_{0}}\right)=\text{dye}\;\;\text{removal}\;\left(\%\right),\\ 100\left(\text{TO}{\text{C}}_{0}-\text{TOC}\right)=\text{TOC}\;\;\text{removal}\;\left(\%\right), \end{array}$$

where *A* and TOC values were received after a certain reaction time and *A*_0_ and TOC_0_ are the initial absorbance and total organic carbon levels.

An additional experiment was carried out to check the possibility of the contribution from a homogenous Fenton reaction, caused by the dissolution of iron from the PA catalyst. In this procedure the same dye solution RB4 (100 mg L^−1^) used in previous experiments was kept in contact with the same PA catalyst (0.2 g L^−1^) at pH 2.5 for 30 minutes. The sample was filtered through a 0.45 *μ*m pore size membrane filter and the (5 mM H_2_O_2_) solution was added for an additional 30 minutes of reaction time. After the reaction time, the sample was filtered through a 0.45 *μ*m pore size membrane filter and analyzed for absorbance and TOC.

## 3. Results and Discussion

### 3.1. Effects of Operational Parameters on Process Efficiency

**Figure 2:**
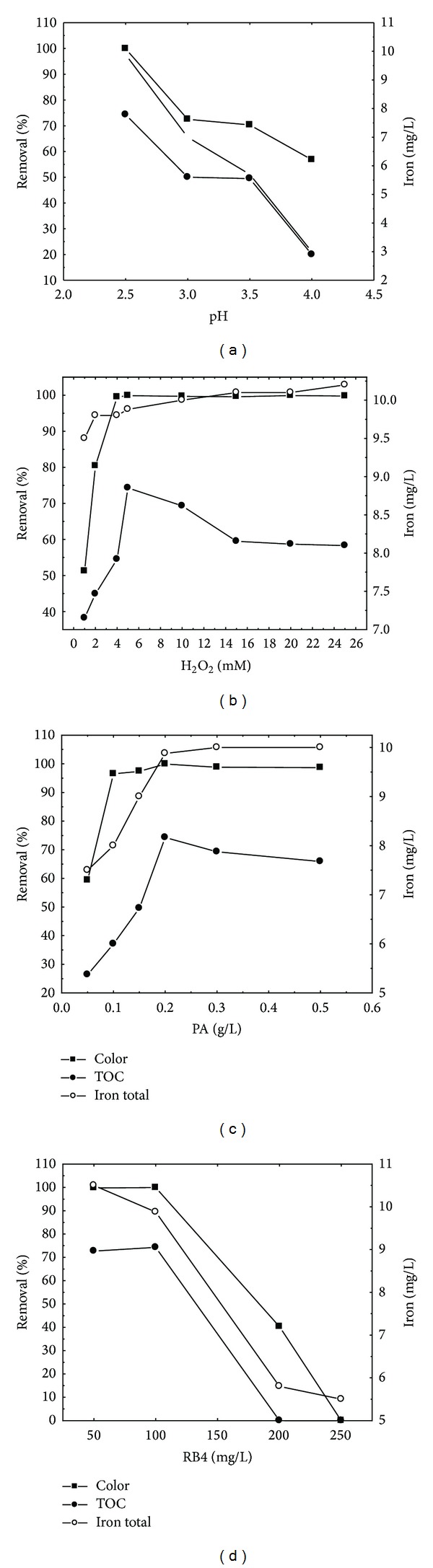
Effects of operational parameters on process efficiency and iron leaching: (a) pH; (b) H_2_O_2_ concentration; (c) PA concentration; (d) RB4 concentration.

The effects of various experimental parameters (solution pH, initial concentration of H_2_O_2_, catalyst (PA), and dye RB4 concentration) are presented in Figure 2.

The classic Fenton process has a typically sharp, preferred pH region in which it is optimally operated. pH affects the activity of both the oxidant and the substrate, the speciation of iron, and hydrogen peroxide decomposition [29]. In this study, the pH effect on the efficiency of solution decolourization was analyzed in the pH range of 2.5–4. The initial concentration of RB4 was 100 mg L^−1^, H_2_O_2_ 5 mM, and PA 0.2 g L^−1^. The maximum colour (99.9%) and TOC (74.3%) removal were obtained at pH 2.5 (Figure 2(a)). This suggests that the PA Fenton reaction also needs the acidic environment (for decolourization efficacy above 90%) required for the proper operation of the classic Fenton system [16] to effectively form ^•^OH and avoid the precipitation of Fe(OH)3_(s)_. Process efficacy dropped with the further increase of the pH values. These results are in accordance with some other studies [7, 30, 31]. It should be noted that at initial pH values as high as 3 to 4, about 60–70% RB4 decolourization is still obtained within the reaction time, which is an improvement over conventional Fenton process [7, 31]. Nevertheless, the solubility of the total iron drops with increasing initial pH. The heterogeneous Fenton process with pyrite ash involves a complex series of reactions on the surface of the catalyst producing ^•^OH and ^•^OH_2_ radicals (reactions 3–6) [25, 31, 32]

(2) $$\begin{array}{c} \text{F}{\text{e}}^{3+}+{\text{H}}_{2}{\text{O}}_{2}⟶\text{F}{\text{e}}^{3+}+\text{O}{\text{H}}^{-}+{\text{H}{\text{O}}_{2}}^{∙}+{\text{H}}^{+} \end{array}$$

(3) $$\begin{array}{c} \text{F}{\text{e}}^{2+}+{\text{H}}_{2}{\text{O}}_{2}⟶\text{F}{\text{e}}^{3+}+\text{O}{\text{H}}^{-}+{\text{HO}}^{∙} \end{array}$$

(4) $$\begin{array}{c} {\text{H}{\text{O}}_{2}}^{∙}⟶{\text{H}}^{+}+{{\text{O}}_{2}}^{∙-} \end{array}$$

(5) $$\begin{array}{c} \text{F}{\text{e}}^{3+}+{\text{H}{\text{O}}_{2}}^{∙}⟶{\text{Fe}}^{2+}+{\text{H}}^{+}+{\text{O}}_{2} \end{array}$$

Pham et al. [33] have also suggested similar mechanisms-including adaptations to the heterogeneous Fenton reaction condition steps based on the original Haber-Weiss mechanism. In those studies, the bottom line is aqueous H_2_O_2_ on the surface of hematite decomposes to H_2_O + O_2_. This may either initiate via a true surface catalytic path the generation of intermediate ^ •^OH or ^•^OH_2_ free radicals or, as we will discuss below, a noncatalytic, nonradical redox route by which solid Fe_2_O_3_ may actually release Fe ions to the solution via a reductive step that leads to aqueous Fe^2+^ (+O_2_). The released aqueous Fe^2+^, together with remaining H_2_O_2_ in the solution, could then fuel a continuing conventional homogeneous Fenton reaction step [25], which can possibly extend the durability of the Fenton process [1, 13, 23, 25].

After 30 min of reaction, the concentration of iron at initial pH values of 2.5 and pH 4 was 9.88 mg L^−1^ and 3 mg L^−1^, respectively. These results indicate a significant effect of pH on the leaching of iron which is supposed to greatly affect the efficiency of the applied process [25]. The increase in oxidation rate at low pH was attributed to the enhanced solubility of iron (III) species at acidic pH, promoting the homogeneous Fenton reaction. Fe(III) can also be solubilised by forming complexes with organic acid intermediates produced during pollutant degradation [34–36]. According to [31] who used the hematite mineral for the decolourization of a reactive dye, the inhibition of iron dissolution can be assigned to the presence of dye molecules which interfere with the interface between the material surface and the aqueous phase.

Colour and TOC removals are enhanced with increasing H_2_O_2_ dosage until reaching the optimum dosage, since it is the source of ^•^OH radicals produced in the Fenton process.  Figure 2(b) shows the dependency of RB4 degradation efficiency on the initial concentration of H_2_O_2_. A high decolourization efficiency, 99.5%, is already achieved with an initial concentration of 4 mM H_2_O_2_. Further increasing the oxidant concentration did not result in a significant effect on the decolourization process. The maximum colour removal was obtained at the initial concentration of 5 mM H_2_O_2_. TOC removal was improved from 38.2 to 74.3% by increasing H_2_O_2_ dose from 1 to 5 mM. With H_2_O_2_ concentrations higher than 5 mM, less mineralization was observed. By following the iron concentration in the solution after the reaction, a mild increase of this metal's content was noted, along with the increase in the initial concentration of H_2_O_2_. Bearing in mind the almost unchanged iron content in all the applied concentrations of H_2_O_2_, it can be concluded that concentrations higher than 5 mM H_2_O_2_ triggered the radical scavenging effect in the solution. Namely, at higher dosages, H_2_O_2_ molecules could trap ^•^OH, thus forming less reactive perhydroxyl radicals (i.e., H_2_O_2_ + ^•^OH → ^•^OH_2_ + H_2_O) and lowering the efficiency of the degradation process [7, 37, 38].

The effect of PA concentration on the efficiency of the process was analyzed within the 0.05–0.5 g L^−1^ range under the following conditions: [H_2_O_2_]_0_ = 5 mM, [RB4]_0 _= 100 mg L^−1^, and pH 2.5 (Figure 2(c)). The Increasing the mass of PA added mass from 0.05 to 0.1 g brought a significant improvement in the decolourization efficiency. It is assumed that the reason for this is the increase of iron content in the Fenton process. This is consistent with literature data [1, 23]. Any further PA concentration increase did not affect the percentage of removed dye in the solution. The maximum decolourization efficiency was achieved with a PA concentration of 0.2 g L^−1^. In mineralization occurrences, adding greater PA amounts than 0.2 g L^−1^ into the solution resulted in a decrease in TOC removals. Some authors [39, 40] explained this phenomenon by naming it a consequence of iron complex formation and some unwanted reactions between iron and other types of formed radicals.

The effect of the initial concentration of RB4 on the decolourization and mineralization was investigated at 50, 100, 200, and 250 mg L^−1^ RB4 (Figure 2(d)). Generally, increasing the dye concentration to values above 100 mg L^−1^ decreased the dye degradation effect. Namely, the increase of the dye concentration leads to an increase in the dye molecules in the solution, with the same number of ^•^OH, which has such an effect as a consequence. With the initial RB4 concentration of 250 mg L^−1^, process has proved completely inefficient. This is accordance with literature data [8, 40]. In heterogeneous Fenton process, the reaction occurs at the catalytic surface between ^•^OH radicals generated at the active sites and RB4 molecules adsorbed on the surface. Thus, when the RB4 concentration is too high, the number of active sites available is decreased by the RB4 molecules due to their competitive adsorption on the catalytic surface. In addition, the intermediates product of RB4 oxidation might also compete for the limited adsorption sites with RB4 molecules, which blocked their interactions with Fe(II)/Fe(III) active sites [8, 30].

The analysis of leached iron dependency on the RB4 dye solution concentration at the end of contact time showed that the iron content in the solution with initial RB4 200 and 250 mg L^−1^ was about 5.5 mg L^−1^ and at the same time was lower than the iron content in the dye solution with initial concentration 100 mg L^−1^. The assumption is that the smaller iron content in the solution contributed to the decreased efficiency of the process in solutions of concentrations higher than 100 mg L^−1^ [1, 16, 23].

It can be concluded that the following reaction conditions give satisfying colour and TOC removal efficiencies in the dye solution with initial concentration up to 100 mg/L: pH = 2.5; [PA]_0_ = 0.2 g/L^−1^; [H_2_O_2_]_0_ = 5 mM.

In order to evaluate the contribution of the homogenous reaction during the overall heterogeneous Fenton reaction, a further experiment was carried out using only the aqueous iron ions produced by PA dissolution at pH 2.5 as the catalyst This experiment showed that the homogenous Fenton reaction resulted in 85% colour removal and 72% TOC removal, respectively. This indicates that most RB4 was degraded by ^•^OH produced from dissolved Fe(II) and not by the surface catalyzed process [23]. This leads to the conclusion that the role of soluble, leached iron from PA is significant in decolourization of RB4.

Thus, at this point of the discussion, it may be considered that the heterogeneous Fenton process is likely to start on the hematite surface by either [25, 31, 32] (a) catalytic decomposition of adsorbed H_2_O_2_ leading to adsorbed ^•^HO and ^•^HO_2_, which, either still on the surface or just after desorption, will attack nearby (adsorbed or not) dye molecules, as proposed by reactions (2) to (5) or (b) as proposed in the present work: with the reduction and release of surface Fe^3+^ as Fe^2+^ into the solution, in which H_2_O_2_ plays the role of reductant. Once in solution, Fe^2+^ will react with the remaining H_2_O_2_, as in the conventional homogeneous Fenton reaction, inducing degradation of the dye molecules. Although not specifically demonstrated in previous works, this hypothesis supports the findings of previous authors [25, 41, 42], who suggested that the heterogeneous Fenton reaction mechanism merely starts on the surface and continues mainly in the bulk of the aqueous solution as a homogeneous Fenton reaction. In those cases, where leaching takes place, overall catalytic activity is a combination of the two types of activities: activity of solid catalysts and activity of dissolved, leached iron, and in further studies, it will be necessary to determine the contribution of these two parameters in the experimental activities.

### 3.2. Solubilisation Rates of Other Metals during the Fenton Reaction

**Figure 3:**
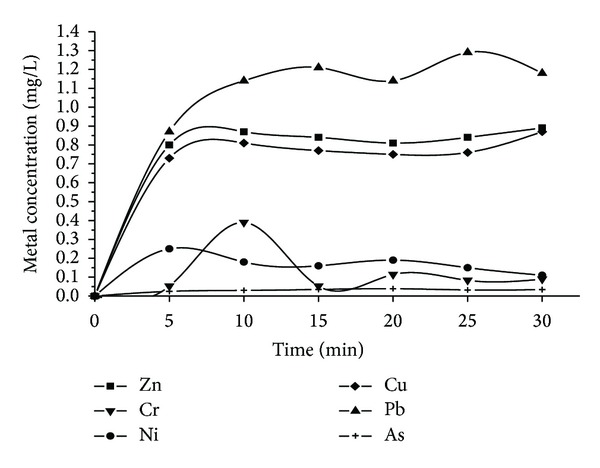
Leaching metals from PA during the reaction (pH = 2.5; [PA]_0_ = 0.2 g/L^−1^; [H_2_O_2_]_0_ = 5 mM; [RB4] = 100 mg/L).

Figure 3 shows solubilisation rates of metals during the reaction time.

The greatest initial metals leaching rates were determined for Pb, Cu, and Zn. This phenomenon can have a positive effect, as transition metals (Cu and Zn), being heterogeneous catalysts, can contribute to the efficiency of the Fenton process [43]. Regardless, applying the PA under optimal conditions for RB4 degradation was satisfactory, but the metals content of the solution, namely, iron, after the process suggests that an additional treatment step is necessary in order to remove the residual metals from the water. Among the principal technologies used and proposed for the removal of potential toxic matters from effluents, precipitation is the most widely used approach for the removal of metals from industrial effluents [44, 45]. Heavy metals, like Cr, Cu, Pb, Mn, and Zn do not precipitate below pH 7.0, allowing their separation from ferric and aluminium ions which precipitate at pH below 6.5 [46, 47].

**Figure 4:**
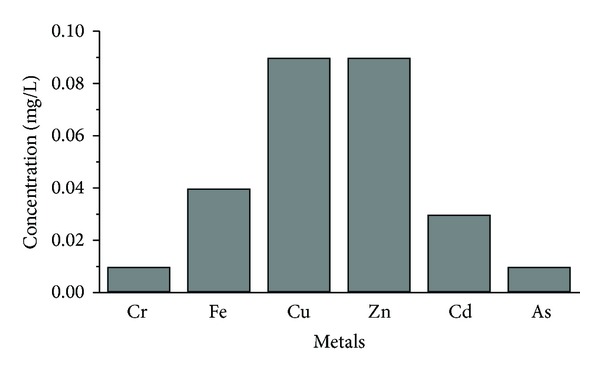
Metal concentrations after lime addition.

Lime was added to the aqueous solution after the Fenton process, until pH 9 was reached. This pH value represents the limit pH value for wastewaters from factories and installations for processing and manufacturing textile, defined by national law [48]. At the same time, this value is close to the pH range 9.5–10.0, which according to [49] presents the pH range of minimal solubility of the hydroxides for the majority of metals. The metal concentrations in the aquatic solutions after the Fenton process and lime addition are presented in Figure 4. The addition of lime in the water solution after the Fenton process contributed to significant reduction in the metals content. The percentage of metal removal from solution ranged from 78 to 99.5%, while Pb and Ni were not detected.

## 4. Conclusion

PA has been proved to be a superior catalyst for decolourization of RB4 in a modified Fenton process. The best operation parameters for the modified Fenton oxidation are 0.2 g L^−1^ of catalyst (PA) and 5 mM of H_2_O_2_, for an initial RB4 concentration of 100 mg L^−1^ at pH 2.5. Under these conditions, 99.9% decolourization and 74.3% mineralization of RB4 in aqueous solution were achieved within a 30 min reaction time.

RB4 dye degradation was satisfactory, but the metal content of the solution after the process suggests that an additional treatment step is necessary to remove the remaining metals from the water. One possible solution is the addition of lime up to pH 9, leading to metal immobilization by forming low solubility hydroxides. On the other hand, lime could also be used as a potential stabilizing agent for the PA remaining after application of the Fenton process.

Due to the large volumes of PA generated, it is necessary to continue rising environmental awareness of different applications for this waste, while taking into account the environmental and economic factors of these waste treatments. Conventional waste management methods, which might have been acceptable in the past, might not be optimal to meet present and future requirements. PA utilisation to “catalyse” a modified Fenton process could be an interesting approach, while taking into account a final treatment for metals in the Fenton process solution and solid residue.

These results provide basic knowledge to better understand the modified, heterogeneous Fenton process and apply the PA Fenton reaction for the treatment of wastewaters characterized by high levels of anthraquinone dyes.

## References

[1] Che H, Bae S, Lee W (2011). Degradation of trichloroethylene by Fenton reaction in pyrite suspension. *Journal of Hazardous Materials*.

[2] Yang C, Chen Y, Peng P, Li C, Chang X, Wu Y (2009). Trace element transformations and partitioning during the roasting of pyrite ores in the sulfuric acid industry. *Journal of Hazardous Materials*.

[3] Pérez-López R, Sáez  R, Álvarez-Valero AM, Nieto JM, Pace G (2009). Combination of sequential chemical extraction and modelling of dam-break wave propagation to aid assessment of risk related to the possible collapse of a roasted sulphide tailings dam. *Science of the Total Environment*.

[4] Oliveiraa MLS, Ward CR, Izquierdo M (2012). Chemical composition and minerals in pyrite ash of an abandoned sulphuric acid production plant. *Science of The Total Environment*.

[5] Gözmen B, Kayan B, Gizir AM, Hesenov A (2009). Oxidative degradations of reactive blue 4 dye by different advanced oxidation methods. *Journal of Hazardous Materials*.

[6] Epolito WJ, Lee YH, Bottomley LA, Pavlostathis SG (2005). Characterization of the textile anthraquinone dye Reactive Blue 4. *Dyes and Pigments*.

[7] Hassan H, Hameed BH (2011). Fe-clay as effective heterogeneous Fenton catalyst for the decolorization of Reactive Blue 4. *Chemical Engineering Journal*.

[8] Lahkimi A, Oturan MA, Oturan N, Chaouch M (2007). Removal of textile dyes from water by the electro-Fenton process. *Environmental Chemistry Letters*.

[9] Arslan-Alaton I, Gursoy BH, Schmidt J-E (2008). Advanced oxidation of acid and reactive dyes: effect of Fenton treatment on aerobic, anoxic and anaerobic processes. *Dyes and Pigments*.

[10] Sadik WA (2007). Effect of inorganic oxidants in photodecolourization of an azo dye. *Journal of Photochemistry and Photobiology A*.

[11] Hammami S, Oturan N, Bellakhal N, Dachraoui M, Oturan MA (2007). Oxidative degradation of direct orange 61 by electro-Fenton process using a carbon felt electrode: application of the experimental design methodology. *Journal of Electroanalytical Chemistry*.

[12] Fu L, You S-J, Zhang G-Q, Yang F-L, Fang X-H (2010). Degradation of azo dyes using in-situ Fenton reaction incorporated into H_2_O_2_-producing microbial fuel cell. *Chemical Engineering Journal*.

[13] Von Sonntag C (2008). Advanced oxidation processes: mechanistic aspects. *Water Science and Technology*.

[14] Karimi A, Aghbolaghy M, Khataee A, Bargh SS (2012). Use of enzymatic bio-Fenton as a new approach in decolorization of malachite green. *The Scientific World Journal*.

[15] Ahmadian M, Reshadat S, Yousefi N (2013). Municipal leachate treatment by Fenton process: effect of some variable and kinetics. *Journal of Environmental and Public Health*.

[16] Che H, Lee W (2011). Selective redox degradation of chlorinated aliphatic compounds by Fenton reaction in pyrite suspension. *Chemosphere*.

[17] Chou S, Huang Y-H, Lee S-N, Huang G-H, Huang C (1999). Treatment of high strength hexamine-containing wastewater by electro-Fenton method. *Water Research*.

[18] Sekaran G, Karthikeyan S, Ramani K, Ravindran B, Gnanamani A, Mandal AB (2011). Heterogeneous Fenton oxidation of dissolved organics in salt-laden wastewater from leather industry without sludge production. *Environmental Chemistry Letters*.

[19] Kušić H, Lončarić Božić A, Koprivanac N, Papić S (2007). Fenton type processes for minimization of organic content in coloured wastewaters. Part II: combination with zeolites. *Dyes and Pigments*.

[20] Kasiri MB, Aleboyeh H, Aleboyeh A (2008). Degradation of Acid Blue 74 using Fe-ZSM5 zeolite as a heterogeneous photo-Fenton catalyst. *Applied Catalysis B*.

[21] Dükkancı M, Gündüz G, Yılmaz S, Yaman YC, Prikhod’ko RV, Stolyarova IV (2010). Characterization and catalytic activity of CuFeZSM-5 catalysts for oxidative degradation of Rhodamine 6G in aqueous solutions. *Applied Catalysis B*.

[22] Wu J, Lin G, Li P, Yin W, Wang X, Yang B (2013). Heterogeneous Fenton-like degradation of an azo dye reactive brilliant orange by the combination of activated carbon-FeOOH catalyst and H_2_O_2_. *Water Science and Technology*.

[23] Bae S, Kim D, Lee W (2013). Degradation of diclofenac by pyrite catalyzed Fenton oxidation. *Applied Catalysis B*.

[24] Zhang A, Wang N, Zhou J, Jiang P, Liu G (2012). Heterogeneous Fenton-like catalytic removal of p-nitrophenol in water using acid-activated fly ash. *Journal of Hazardous Materials*.

[25] Garrido-Ramírez EG, Theng BKG, Mora ML (2010). Clays and oxide minerals as catalysts and nanocatalysts in Fenton-like reactions—a review. *Applied Clay Science*.

[26] Microwave assisted acid digestion of sediments, sludges, soils and oils.

[27] Flame atomic absorption spectrophotometry.

[28] Graphite Furnace Absorption Spectrophotometry.

[29] Zhang H, Choi HJ, Huang C-P (2005). Optimization of Fenton process for the treatment of landfill leachate. *Journal of Hazardous Materials*.

[30] Chen A, Ma X, Sun H (2008). Decolorization of KN-R catalyzed by Fe-containing Y and ZSM-5 zeolites. *Journal of Hazardous Materials*.

[31] Araujo FVF, Yokoyama L, Teixeira LAC, Campos JC (2011). Heterogeneous Fenton process using the mineral hematite for the discolouration of a reactive dye solution. *Brazilian Journal of Chemical Engineering*.

[32] Kwan WP, Voelker BM (2003). Rates of hydroxyl radical generation and organic compound oxidation in mineral-catalyzed fenton-like systems. *Environmental Science and Technology*.

[33] Pham AL-T, Lee C, Doyle FM, Sedlak DL (2009). A silica-supported iron oxide catalyst capable of activating hydrogen peroxide at neutral pH values. *Environmental Science and Technology*.

[34] Feng J, Hu X, Yue PL (2006). Effect of initial solution pH on the degradation of Orange II using clay-based Fe nanocomposites as heterogeneous photo-Fenton catalyst. *Water Research*.

[35] Martínez CE, McBride MB (2000). Aging of coprecipitated Cu in alumina: changes in structural location, chemical form, and solubility. *Geochimica et Cosmochimica Acta*.

[36] Bobu M, Yediler A, Siminiceanu I, Schulte-Hostede S (2008). Degradation studies of ciprofloxacin on a pillared iron catalyst. *Applied Catalysis B*.

[37] Behnajady MA, Modirshahla N, Ghanbary F (2007). A kinetic model for the decolorization of C.I. Acid Yellow 23 by Fenton process. *Journal of Hazardous Materials*.

[38] Joseph JM, Destaillats H, Hung H-M, Hoffmann MR (2000). The sonochemical degradation of azobenzene and related azo dyes: rate enhancements via Fenton's reactions. *The Journal of Physical Chemistry A*.

[39] Carriazo J, Guélou E, Barrault J, Tatibouët JM, Molina R, Moreno S (2005). Synthesis of pillared clays containing Al, Al–Fe or Al–Ce–Fe from a bentonite: characterization and catalytic activity. *Catalysis Today*.

[40] De Laat J, Le TG (2006). Effects of chloride ions on the iron(III)-catalyzed decomposition of hydrogen peroxide and on the efficiency of the Fenton-like oxidation process. *Applied Catalysis B*.

[41] Teel AL, Warberg CR, Atkinson DA, Watts RJ (2001). Comparison of mineral and soluble iron Fenton's catalysts for the treatment of trichloroethylene. *Water Research*.

[42] Lu M-C, Chen J-N, Huang H-H (2002). Role of goethite dissolution in the oxidation of 2-chlorophenol with hydrogen peroxide. *Chemosphere*.

[43] Pirkanniemi K, Sillanpää M (2002). Heterogeneous water phase catalysis as an environmental application: a review. *Chemosphere*.

[44] Mirbagheri SA, Hosseini SN (2005). Pilot plant investigation on petrochemical wastewater treatment for the removal of copper and chromium with the objective of reuse. *Desalination*.

[45] Levasseur B, Blais J-F, Mercier G (2005). Study of the metal precipitation from decontamination leachates of municipal wastes fly ash incinerators. *Environmental Technology*.

[46] McBride MB, Martínez CE (2000). Copper phytotoxicity in a contaminated soil: remediation tests with adsorptive materials. *Environmental Science and Technology*.

[47] Lee G, Bigham JM, Faure G (2002). Removal of trace metals by coprecipitation with Fe, Al and Mn from natural waters contaminated with acid mine drainage in the Ducktown Mining District, Tennessee. *Applied Geochemistry*.

[48] Ministry of Energy, Development and Environmental Protection of Republic of Serbia (2011). Regulation on limit values of pollutants in water and deadlines for their achievement. *Official Gazette*.

[49] Chen Q, Luo Z, Hills C, Xue G, Tyrer M (2009). Precipitation of heavy metals from wastewater using simulated flue gas: sequent additions of fly ash, lime and carbon dioxide. *Water Research*.

